# Permutational analysis of *Saccharomyces cerevisiae* regulatory elements

**DOI:** 10.1093/synbio/ysaa007

**Published:** 2020-06-16

**Authors:** Namrita Dhillon, Robert Shelansky, Brent Townshend, Miten Jain, Hinrich Boeger, Drew Endy, Rohinton Kamakaka

**Affiliations:** y1 Department of MCD Biology, University of California, Santa Cruz, CA, USA; y2 Department of Bioengineering, Stanford University, Stanford, CA, USA; y3 Department of Biomolecular Engineering, University of California, Santa Cruz, CA, USA

**Keywords:** *Saccharomyces cerevisiae*, gene activation, transcription, synthetic biology

## Abstract

Gene expression in *Saccharomyces cerevisiae* is regulated at multiple levels. Genomic and epigenomic mapping of transcription factors and chromatin factors has led to the delineation of various modular regulatory elements—enhancers (upstream activating sequences), core promoters, 5′ untranslated regions (5′ UTRs) and transcription terminators/3′ untranslated regions (3′ UTRs). However, only a few of these elements have been tested in combinations with other elements and the functional interactions between the different modular regulatory elements remain under explored. We describe a simple and rapid approach to build a combinatorial library of regulatory elements and have used this library to study 26 different enhancers, core promoters, 5′ UTRs and transcription terminators/3′ UTRs to estimate the contribution of individual regulatory parts in gene expression. Our combinatorial analysis shows that while enhancers initiate gene expression, core promoters modulate the levels of enhancer-mediated expression and can positively or negatively affect expression from even the strongest enhancers. Principal component analysis (PCA) indicates that enhancer and promoter function can be explained by a single principal component while UTR function involves multiple functional components. The PCA also highlights outliers and suggest differences in mechanisms of regulation by individual elements. Our data also identify numerous regulatory cassettes composed of different individual regulatory elements that exhibit equivalent gene expression levels. These data thus provide a catalog of elements that could in future be used in the design of synthetic regulatory circuits.

## 1. Introduction

Gene expression in eukaryotes is regulated at multiple levels, including transcriptional, post-transcriptional and translational control. Mutational analysis as well as genomic and epigenomic mapping of proteins have led to the definition and delineation of modular sequence elements defining enhancers, promoters, 5′ and 3′ untranslated regions (UTRs) as well as transcriptional terminators. The 5′ UTR is involved in the association of the messenger ribonucleicacid (mRNA) with the ribosome while the 3′ UTR is involved in mRNA stability and turnover. Well-defined upstream activating enhancer sequences (UAS) direct the initiation of transcription in response to signals while modular promoter sequences made up of the TATA box and initiator elements function as sites of binding for the general transcription factors and RNA polymerase II. Often the promoter and UAS enhancers are conflated together but, in this article, we will use the term core promoter to refer to the deoxyribonucleic acid (DNA) elements that are bound by the general transcription factors and the polymerase and the term enhancer to refer to the UAS (1). 

Gene expression involves the integration of numerous different signals affecting different regulatory elements. Regulation is mediated by sequence-specific binding proteins that recognize their cognate binding sites in DNA and RNA. While the protein-bound elements are modular and interchangeable, they do not function in isolation. Thus, expression ultimately involves effective integration of all of the signals via functional communication between different regulatory elements leading to a defined output.

Early studies on gene regulation investigated regulatory elements of single genes via directed mutagenesis of sequences (2–4). These analyses gave way to saturation mutational studies of a single well-defined element, such as an enhancer, against a background of other elements being left unchanged (5). While very valuable, these approaches did not systematically study the ability of an element to functionally communicate with other elements in a regulatory cassette.

Since there are many possible functional interactions between regulatory elements, characterizing one element at a time is not feasible given the large numbers of permutations possible. An alternative approach to investigate the complex regulatory landscape was developed to study the functional interactions between promoters and 5′ UTRs in prokaryotes (6–8). The method involved constructing a large permutational library of these elements controlling the expression of a fluorescent reporter and introducing them into cells which were then sorted on the basis of the expression levels of the fluorescent reporter. The identity of the regulatory element mediating a specific level of expression was determined by high-throughput sequencing of the sorted subpopulations of cells with defined expression levels. The highly parallelized experiments using a combinatorially rich set of reporters permitted the study of large numbers of regulatory elements and their influence on each other.

We have chosen to develop a similar approach to characterize regulatory elements in the eukaryote *Saccharomyces cerevisiae*. We initially delineated the regulatory space of 26 different yeast genes into four regulatory elements: UAS enhancers, core promoters, 5′ UTRs and 3′ UTR/terminator based on published databases. We cloned these elements using the recently developed modular cloning kit (9). We then used a directed Golden Gate ligation approach to rejoin the fragments in the correct order thus creating complete synthetic genes albeit with regulatory parts chosen at random from the 26 different genes. This 400 000 cassette permutational library was transformed into yeast cells and the cells were fractionated using flow cytometry and cell sorting on the basis of expression levels of a fluorescent reporter gene. The reporter gene with its associated regulatory elements from the fractionated cells were sequenced using next-generation nanopore sequencing to identify the regulatory elements that played a role in directing a specific level of gene expression. We validated this approach by building a directed matrix where nine representative UAS enhancers were combined with nine different core promoters to study the functional communication between these elements. This 81-cassette matrix also allowed us to investigate how the different elements influence each other under varying environmental and mutagenic conditions.

## 2. Results 

### 2.1 Fragmentation of the regulatory space and combinatorial library preparation

The standardization of biological parts and their characterization under varying growth, environmental and genetic backgrounds is necessary in order for these parts to be routinely mixed and matched for use in synthetic circuits (10). Transcription of protein-coding genes in yeast is mediated by regulatory sequences located upstream and downstream of the gene. To initiate this study, we initially chose ∼60 different yeast genes based simply on their expression levels in glucose (11–13). From the initial 60 genes we picked 26 genes for experimental analysis based on the availability of data mapping various epigenetic marks and transcription factors. Approximately a quarter of these 26 genes are not active in glucose-containing media while the rest are expressed to varying degrees.

**Figure 1.**
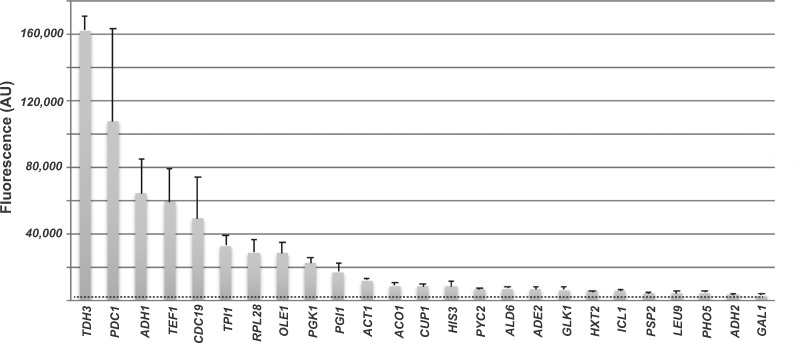
Expression profiles of native regulatory elements. The native UAS enhancer, core promoter and 5′ UTR of different genes were fused to a Venus reporter gene and a *PGK1* 3′ UTR. Expression of the Venus reporter, measured using a fluorometer, was plotted.

To validate published expression data for the 26 chosen genes, we built constructs where the endogenous UAS enhancer–core promoter–5′ UTR for these 26 genes were fused to the coding region of the fluorescent Venus protein along with the *PGK1* 3′ UTR/transcription terminator. These constructs were transformed into yeast cells and the level of expression of Venus in glucose-rich YMD media was measured using a fluorescent plate reader. The data were plotted and show a continuum of expression values with the *TDH3* regulatory sequences generating the most fluorescence while the inducible genes produced the least fluorescence (Figure 1).

We next demarcated the regulatory space of these genes using specific databases. We identified the functional 3′ UTR and transcription terminator sequences of the 26 genes based on the transcription terminator dataset (14). We identified the start of transcription for these genes based on RNA-seq data of transcripts generated in glucose (11–13). Sequences downstream of the transcription start site and up to the start codon were delineated as 5′ UTRs. The core promoter was designated as a TATA binding protein (TBP) bound nucleosome-free region (NFR) containing fragment (12, 15–18). Approximately half of the genes chosen have a TATA box while the rest are considered ‘TATA-less’ or were unannotated (12). The chromatin architecture of the upstream regulatory regions of these 26 different genes was mapped using ATAC-seq data (19). These accessible sites have previously been shown to occur at protein binding sites in chromatin (Supplementary Figure S1).

**Figure 2.**
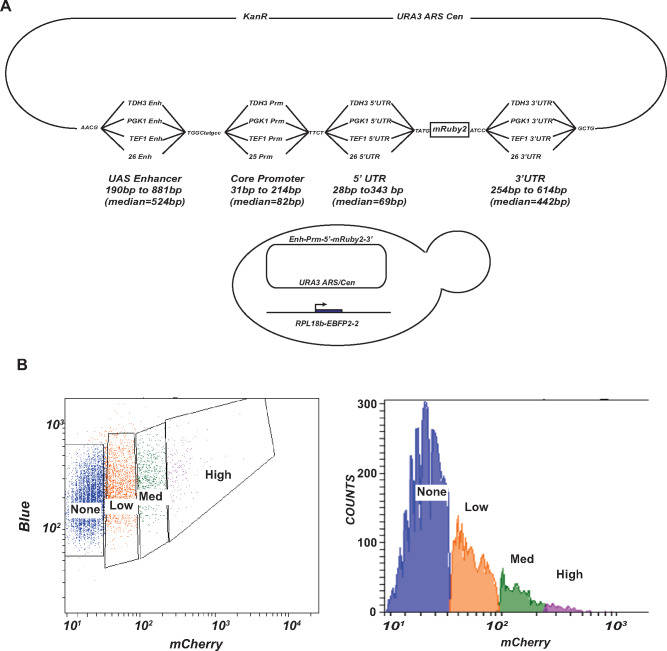
Library construction and FACS. (**A**) Schematic representation of a library constructed from permutations of different regulatory elements driving expression of the mRuby2 reporter. The library was transformed into yeast cells expressing EBFP2-2 under the control of the *RPL18b* promoter. (**B**) Cytometry traces and sorted bins of yeast cells transformed with the permutation library. Cells with no, low, medium and high mRuby2 expression were collected in four fractions. Blue: no mRuby2 expression, orange: low mRuby2 expression, green: medium mRuby2 expression and purple: high mRuby2 expression. Each sorted fraction had a different number of cells.

Each of the delineated regulatory fragments (UAS enhancer, core promoter, 5′ UTR and 3′ UTR) from the 26 genes were polymerase chain reaction (PCR) amplified using specific primers (Supplementary Table S1) and cloned into the recently described parts vector pYTK001 (9). The parts plasmids were then used to create a permutational library (using a Golden Gate ligation protocol) such that the fragments would combine in a directed but random manner (9). This resulted in ∼400 000 recombinant plasmids containing different permutations of the four regulatory elements, UAS enhancer, core promoter, 5′ UTR and 3′ UTR, controlling the expression of a fluorescent reporter, mRuby2 (Figure 2A).

The purified library was transformed into W-303 yeast cells (ROY5634). This strain also contained a fluorescent protein mTagEBFP2-2 under the control of the *RPL18b* promoter. Cells containing the library were grown to log phase in glucose-containing media and sorted using a fluorescence-assisted cell sorter (FACS) based on the expression of both mRuby2/EBFP2-2. We gated the library into four expression fractions (no expression, low, medium and high expression) (Figure 2B). The gates for fluorescent cell sorting were based on various control strains (see Supplementary Figure S2 and ‘Materials and methods’ section). Sixty-three percent of the sorted cells were in the no expression sorted fraction, 26% were in the low expression fraction, 8% were in the medium expressing fraction and 3% were in the high expressing fraction.

DNA was isolated from the four sorted pools and the entire expression cassette, including mRuby2, was PCR amplified. Barcodes were ligated to the amplified fragments to distinguish the four sorted pools and the PCR products were subsequently sequenced using an Oxford Nanopore MinION sequencer. Of the total mapped reads, 61% were from the no mRuby2 expression fraction, 19% were from the low expression fraction, 5% were from the medium mRuby2 expression fraction and 14% were from the high mRuby2 expressing fraction.

### 2.2 Extent of expression mediated by specific elements

**Figure 3.**
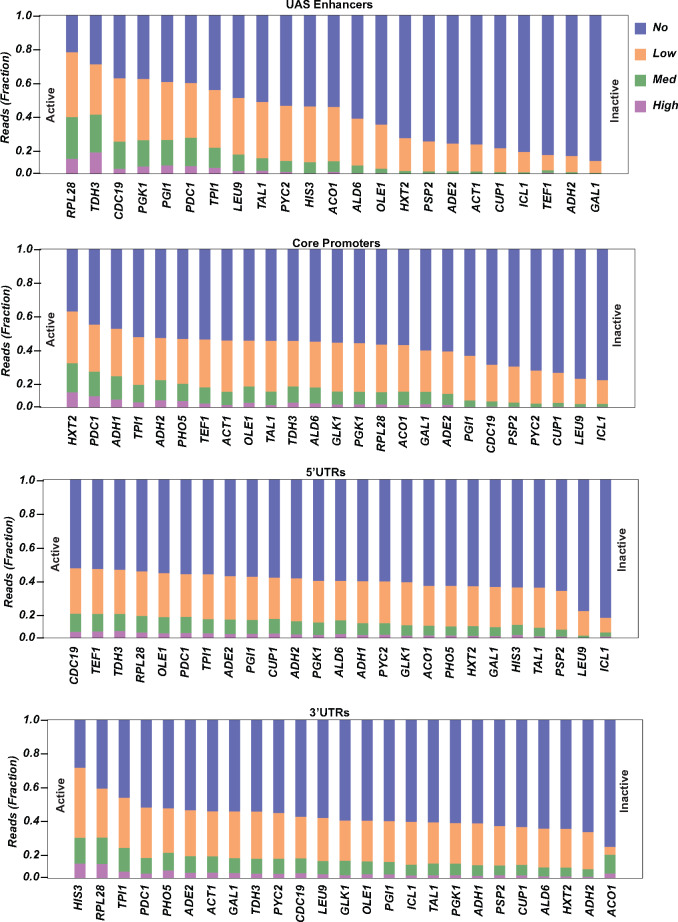
Stacked histograms of the percent of each regulatory element (UAS enhancer, core promoter, 5′ UTR and 3′ UTR/terminator) present in each of the four sorted fractions. The elements are rank-ordered based on mRuby2 expression. Blue bars represent the non-expressing fraction, orange bars represent the low-expressing fraction, green bars represent the medium expressing fraction and purple bars represent the high expressing fraction.

The premise of the approach is that the relative distribution of specific regulatory elements in the sorted cell fractions is a function of the level of expression of the mRuby2 reporter gene. We determined the ratio of a specific regulatory element in each sorted cell fraction by estimating the number of cells observed for each regulatory element in each cell fraction as described in ‘Materials and methods’ section. We plotted the distribution of the elements across the four sorted fractions as stacked histograms and rank-ordered the elements based on their expression levels (Figure 3).

When one focuses on the distribution of UAS enhancers in the four sorted fractions, the UAS enhancers for the inducible genes *GAL1, ADH2, CUP1* and *ICL1* are present almost exclusively in the non-expressing fraction. This is consistent with the fact that these genes are inactive in glucose-rich media. On the other hand, the *TDH3* and *RPL28* UAS enhancers are enriched in the high and medium expressing fractions, this is also consistent with expression analysis of yeast cells growing in glucose-rich media (11–13).

The same analysis performed with the core promoter fragments finds a striking discordance. The genes *PGI1* and *CDC19* are both highly expressed in glucose-containing media (11–13), but their core promoter fragments are present to a greater extent in the non-expressing fractions. In addition, core promoters of genes not active in glucose-containing media such as *HXT2, PHO5* and *ADH2* are present to a greater extent in the highly expressing fractions. With respect to the UTRs, the 26 different UTR fragments have similar distributions across the four sorted fractions, with a few exceptions, suggesting that they play a lesser role in regulating levels of gene expression.

**Figure 4.**
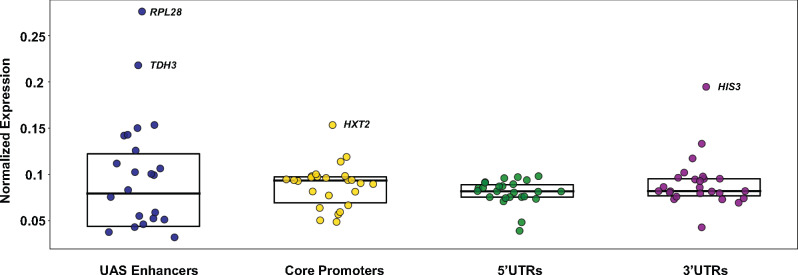
Expression analysis of each regulatory element. The mean expression of a regulatory fragment was generated by taking a weighted average of the fluorescence for each expression category (determined by FACS) times the number of reads for each fragment within each of the four sorted fractions. Box plots depicting the normalized expression of all 26 regulatory elements are shown. The box represents the 25th to 75th percentiles and the line across the box represents the median.

We next calculated the mean expression exhibited by each regulatory element by fitting an estimate of cell counts for each regulatory fragment across each sorted fraction to a log-normal distribution as described in ‘Materials and methods’ section. Thus, the minimum expression that could be achieved by an enhancer would occur if that enhancer was solely present in the no expression FACS pool. Similarly, maximum expression by an enhancer would be achieved if that enhancer was solely present in the highly expressing FACS pool. These values were plotted as box plots where each dot represents the regulatory element from one of the 26 different genes (Figure 4). The box plots show that the different UAS enhancers affect expression over a large range, with inducible enhancers (*GAL1*) being inactive in glucose-rich media and the glucose-induced and housekeeping enhancers (*TDH3* and *RPL28*) being active to varying extents under these growth conditions. The core promoter fragments affect expression levels as well, but the spread in expression levels is less than that observed with the enhancers and a similar profile is seen with the 3′ UTRs. On the other hand, most of the 5′ UTRs cluster together indicating that the different 5′ UTRs function more or less equivalently.

It should be noted that in this study we are measuring protein levels and the fold difference between the highly expressed genes and the inactive genes is not as great as the fold difference reported for these genes via measurements of mRNA. This could be due to protein homeostasis dampening the expression levels of mRuby2 mediated by the very strong UAS enhancers.

### 2.3 Pairwise interactions between regulatory elements

**Figure 5.**
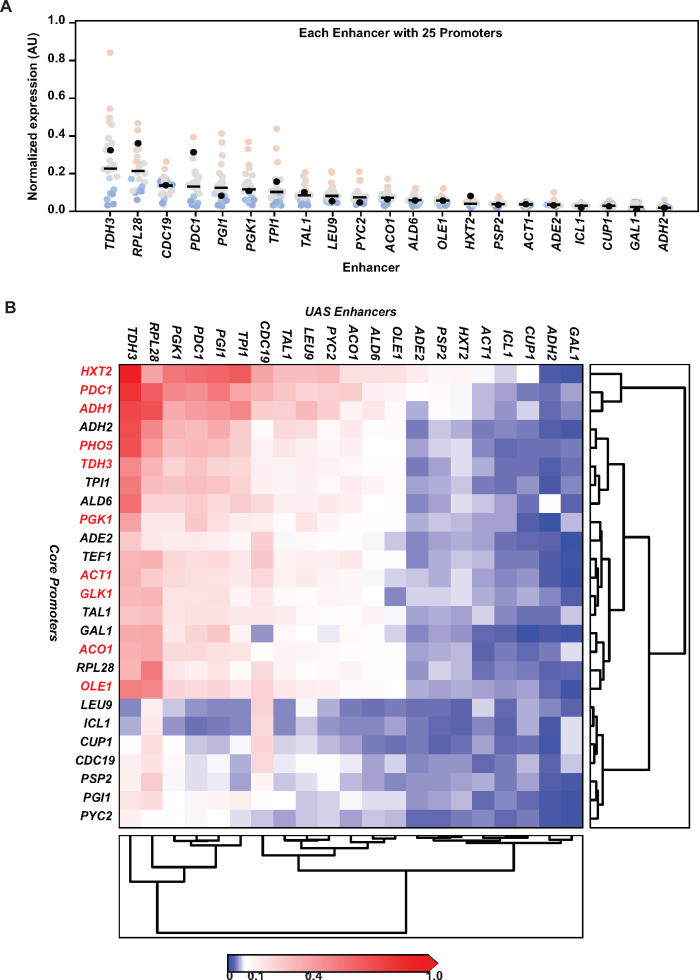
Expression analysis of pairwise combinations of regulatory elements. (**A**) The mean expression of a regulatory fragment was calculated by taking a weighted average of the fluorescence for each expression category (determined by FACS) times the number of reads of each fragment within each of the four sorted bins. Pairwise comparison of enhancers with promoters is shown as box plots. Each enhancer is shown on the *X*-axis. Each dot in the box plot represents one specific core promoter element. The black dot represents the expression level mediated by the enhancer in combination with its native promoter. (**B**) Heat map and clustering to identify relationships between pairs of UAS enhancers and core promoters. The calculated expression values of each pairwise combination were plotted as a heat map and the values were used to generate clusters based on expression levels. TATA-containing core promoters are labeled in red.

There are many idiosyncratic and context-dependent interactions between regulatory elements. Trying to identify these functional interactions by characterizing one pair of regulatory elements at a time is not practical given the large numbers of permutations that are possible and that would need to be tested. However, the data from the sorted permutational library allow us to analyze all possible pairwise interactions. We therefore calculated the expression patterns mediated by pairs of regulatory elements calculated as above (while ignoring the contributions from the other regulatory elements). For example, expression values mediated by each UAS enhancer fragment when paired with one of the 25 different core promoters were determined. These data were then plotted as a box plot (Figure 5A) with each of the 25 different promoters being represented as a single dot; the black dot is the enhancer with its native promoter. The ordering of the enhancers based on their mean expression levels identifies strong, intermediate and inactive enhancers in glucose-containing media. This rank distribution is consistent with published measurements of mRNA levels from these genes (11–13) but there are some differences in the ranking presumably because the other regulatory elements alter enhancer-mediated expression levels.

The same data were also transformed into heat maps (Figure 5B). These data highlight an interesting role for promoters in mediating gene activity levels. The *TDH3* gene is one of the most highly expressed genes in yeast. When we analyzed the *TDH3* UAS enhancer with the core promoters we found several core promoters (*HXT2, PDC1, ADH1*, etc.) that were able to increase gene expression over the native *TDH3* enhancer–promoter pair. Similar increases in expression were observed for the other active enhancers as well. This change in expression was not only in one direction. Several core promoters dampened expression from even the strongest enhancers. For instance, the *TDH3* UAS enhancer-mediated expression was significantly reduced by the *LEU9*, *CUP1* and *ICL1* core promoters. This suggests that core promoters functionally communicate with UAS enhancers to modulate the levels of UAS enhancer-mediated transcription.

One of the goals of this massively parallel approach is to identify unique combinations of regulatory elements that generate equivalent levels of expression. Presenting the data as heat maps quickly identifies and highlights different combinations of elements that mediate similar levels of expression. For example, the *TDH3* UAS enhancer generates very similar levels of expression when paired with either *PDC1*, *ADH1*, *ADH2* or *PHO5* core promoters. Similar equivalent expression patterns are observed for other combinations of elements as well (Figure 5B). These data should therefore provide a useful resource for individuals wishing to achieve a specific level of gene activity with different combinations of regulatory elements. Biochemical and molecular analysis have previously been used to study the determinants of UAS enhancer-mediated gene activation (20) and similar studies in future could be used to investigate how these novel combinations of regulatory elements mediate specific expression levels from these synthetic cassettes.

Pairwise heat maps can also be clustered using hierarchical clustering methods based on weighted expression levels. This clustering can be used to classify and sub-categorize different regulatory elements. The clustering data for UAS enhancers identified three main sub-clusters—a high and a low expressing cluster and a cluster where the UAS enhancer is not active (Figure 5B). The clustering data, though not robust, also suggest that ‘TATA-less’ core promoters are weak promoters while the TATA-containing core promoters cluster to some extent as strong promoters which is consistent with studies of core promoters in yeast and human cells (21).

We also asked if there was any correlation between enhancer strength and promoter strength. Our data show that there was no correlation between the rank order of enhancer activity and the rank order of promoter activity. This is likely due to the fact that the inducible genes we analyzed here are expected to be inactive in glucose media. When their native promoters are separated from their cognate enhancers and the promoters are ectopically paired with other enhancers, then these promoters’ innate ability to foster high expression manifests itself. It is for this reason that *HXT2* and *ADH2* UAS enhancers are inactive but their core promoters are among the strongest.

### 2.4 Principal component analysis highlights regulatory element outliers

**Figure 6.**
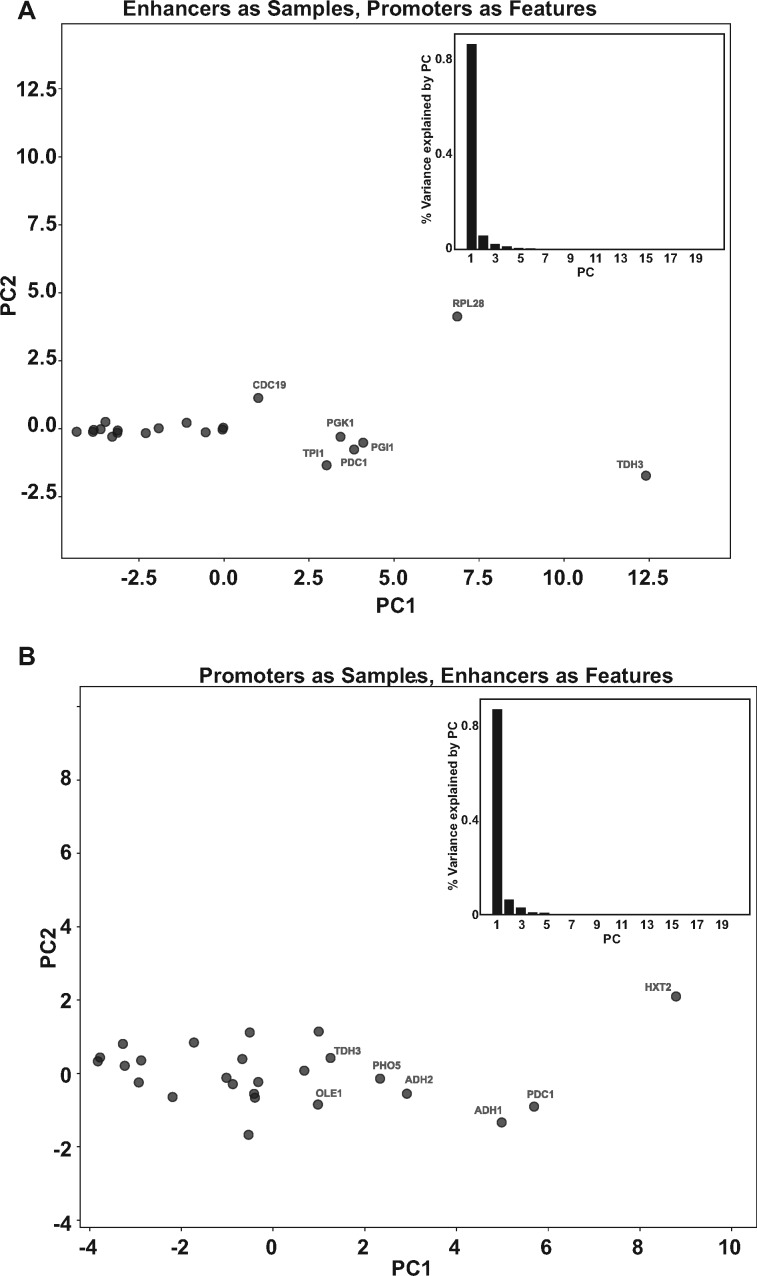
PCA of pairwise combinations of regulatory elements. (**A**) The score plot displays each sample with respect to the first two principal components with UAS enhancers as samples and core promoters as features and was used to determine the relationship among the samples. The percent variance present within each principal component is plotted in the inset. (**B**) The score plot displays each sample with respect to the first two principal components with core promoters as samples and UAS enhancers as features and was used to determine the relationship among the samples. The percent variance present within each principal component is plotted in the inset.

Principal component analysis (PCA) is useful in identifying the axes of observed variation in datasets. Our expression dataset for the regulatory elements indicated that the principal driver of differences in expression levels were the UAS enhancer and the core promoter. Each of these elements could affect expression levels via multiple and different means. Using our data, we sought to identify the number of principal means by which UAS enhancers affected gene expression levels. A PCA using enhancers as samples and promoters as features, effectively distributed enhancers in promoter space (Figure 6A). This analysis found that one predominant axis of variation across the 26 different core promoters explained ∼90% of the total variance (Figure 6A, inset graph). Plotting the 26 enhancers across the first two principal components shows no distinct clusters, rather a gradient emerges. This distribution mirrors the rank order of these UAS enhancers based on expression levels. We therefore infer that PC1 reflects the ability of an enhancer to amplify expression, UAS enhancer strength. Since similarities in the data are correlated with distance in the projection space (defined by the PCs), the analysis also allows us to identify outliers. The *RPL28* enhancer occupies a distinct position on PC2, while *TDH3* is an outlier along PC1 demonstrating its ability to mediate very high levels of expression.

The same analysis with promoters as samples and enhancers as features also showed that one principal component explains ∼90% of the total variance (Figure 6B, inset graph) and PC1 likely reflects promoter strength, with ‘TATA-less’ core promoters at one end and strong TATA-containing promoters at the other. Interestingly, PC1 for 5′ UTRs and 3′ UTR’s (data not shown) explains only ∼60% of the total variance suggesting a more complex regulatory relationship between the UTRs and the other elements though the exact nature of this variation remains to be determined.

### 2.5 9 × 9 Matrix of enhancers and promoters

**Figure 7.**
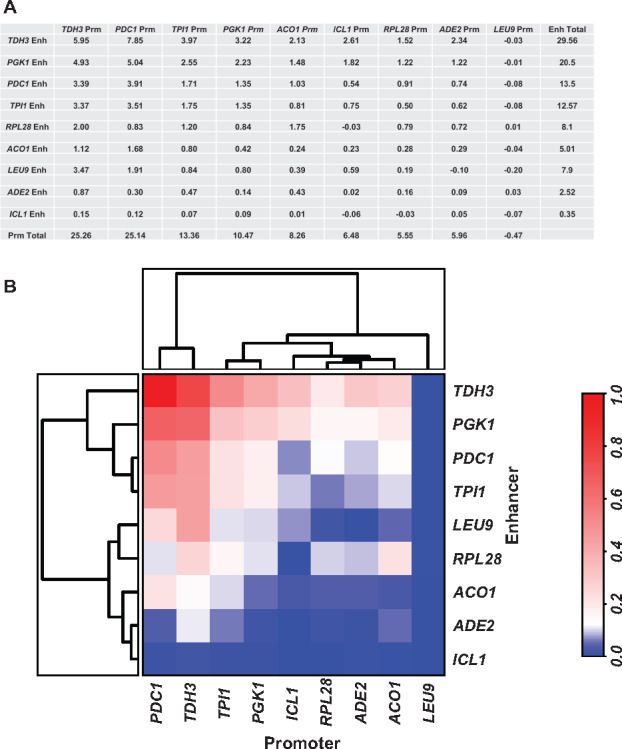
Expression analysis of a 9** × **9 matrix of UAS enhancers with core promoters + 5′ UTRs in glucose containing medium. (**A**) A 9** × **9 matrix of different combinations of UAS enhancers and core promoters + 5′ UTRs with the Venus reporter gene and the *PGK1* 3′ UTR was generated. Venus expression of these constructs was measured using a fluorometer. The expression of each individual pairwise combination was listed as a percentage of the sum of the expression values of all 81 constructs. The mean values of three biological replicates of each construct are presented. (**B**) The Venus expression values of pairwise combinations (UAS enhancers and core promoters + 5′ UTR) were plotted as a heat map along with clustering analysis to identify relationships between elements.

**Figure 8.**
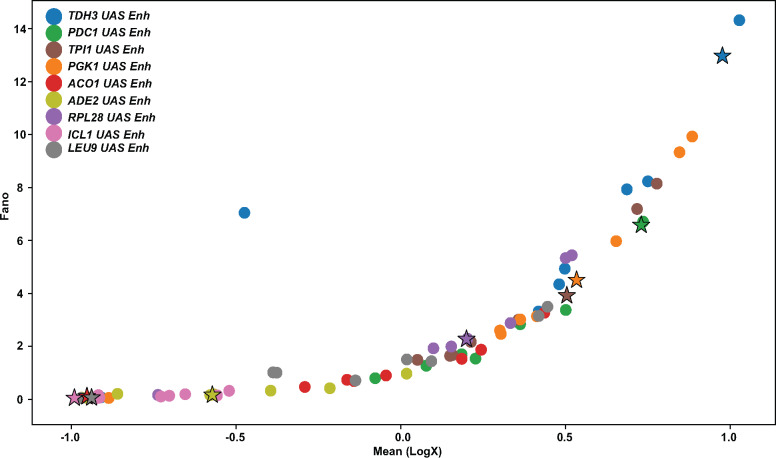
Cytometry of the 9** × **9 combination of enhancers with promoters measuring Venus expression and variation in expression. Cells containing the 81 constructs were grown to log phase and analyzed in a flow cytometer to measure expression levels in individual cells as well as the level of variation. Three biological replicates were performed for each construct. A plot of Fano factor against mean expression is shown.

The random combinatorial approach involving cell sorting and next-generation sequencing is powerful, but it is not easily amenable for use with different environmental conditions or in combinations with various mutants. To validate the results obtained by FACS and nanopore sequencing and to study the effects of various enhancer–promoter combinations on responses to varying growth conditions, we selected nine genes that span different expression levels. We systematically built a matrix of 81 different constructs with the UAS enhancers combined with the core promoter and 5′ UTRs from these 9 genes and each construct controlled the expression of a Venus reporter gene. The *PGK1* 3′ UTR was used in all of these constructs. These 81 constructs were transformed into yeast cells and the cells were arrayed as a 9** × **9 matrix. We measured the expression of the fluorescent Venus reporter using a fluorescent plate reader. The fluorescence intensity (expressed as a percent of the total) was normalized to the total intensity observed across the sum of all 81 constructs (Figure 7A). This analysis presents the regulatory strength of each combinatorial cassette as a fraction of the total expression space. This approach allows us to analyze data from different fluorimeters with different sensitivities as well as data collected on different days under slightly different growth conditions. Each experiment was repeated between three and seven times (Figures 7 and 8).

In glucose-containing media, the *PGK1* enhancer with its cognate promoter generates 2.23% of the total fluorescence. This value almost doubles when the *PGK1* UAS enhancer is combined with either the *TDH3* or the *PDC1* core promoters (Figure 7A). Similarly, the *TDH3* UAS enhancer with its cognate promoter generates ∼5.95% of the total fluorescence landscape. This value increases to 7.85% when the *TDH3* enhancer is combined with the *PDC1* core promoter. Other promoters are unable to increase expression from these strong UAS enhancers and actually reduce expression. For example, the *TDH3* UAS enhancer in combination with the *PGK1* core promoter only accounts for 3.22% of the total fluorescence. In comparison to the very strong enhancers, analysis of moderately strong enhancers shows vast increases in expression potential when these enhancers are combined with core promoters from other active genes. For example, the *ACO1*, *RPL28* and *TPI1* enhancers–promoter combinations generate high levels of transcripts but the levels can be increased significantly by swapping their native promoters with the promoters of the strong genes. These data suggest that while cognate promoters are optimized to work with their native UAS enhancers, optimization does not imply maximizing expression, there exists an ability to significantly increase or decrease expression when these enhancers are combined with other core promoters.

Using these data, we determined the enhancer and promoter strengths for all nine genes and plotted these as heat maps (Figure 7B). Both enhancer and promoter elements positively and negatively influence expression. For example, the native *TDH3* and *PDC1* cassettes are ranked first and second in overall expression (Figure 1). However, when the UAS enhancer and promoter are separated, the *PDC1* promoter increases expression from the *TDH3* enhancer while the *TDH3* promoter dampens expression from the *PDC1* enhancer (Figure 7B) and the highest expressing cassette is the *TDH3* enhancer combined with the *PDC1* promoter. Similarly, the native *ICL1* cassette is inactive in glucose-rich media. However, analyzing its promoter separated from its native UAS enhancer and combined with the enhancers of other genes, the *ICL1* promoter is a moderately strong promoter.

### 2.6 Total noise scales with mean expression levels

The relationship between expression levels and total noise is partly dependent upon promoter and enhancer architecture and chromatin configuration (22). We therefore investigated the gene expression of the 81-cassette matrix using cytometry (Figure 8). Cytometry allows us to measure transcription from the expression cassette in single cells and thus allows us to measure the variation in expression across cells with the same genetic background. Yeast cells containing the expression cassettes were analyzed using a fluorescent cytometer to measure the level of expression of the Venus reporter. These experiments were done in triplicate and there is a good correspondence between the three replicates (data not shown). The mean fluorescence and the Fano factor values were calculated. The data show that the Fano factor was significantly greater than one for all of the constructs measured. We next analyzed the relationship between the Fano factor and the mean expression. There was an increase in Fano factor with a corresponding increase in the mean and the relationship appears to track a universal curve. The universal relationship could be caused by a collection of different noise sources, both biological and technical but the data are consistent to what has been observed before (23–27). Since we tested TATA-containing and TATA-less promoters, our data indicate that the relationship is not simply dependent on the presence or absence of a TATA box.

### 2.7 Measurements of expression as a function of different environmental conditions

**Figure 9.**
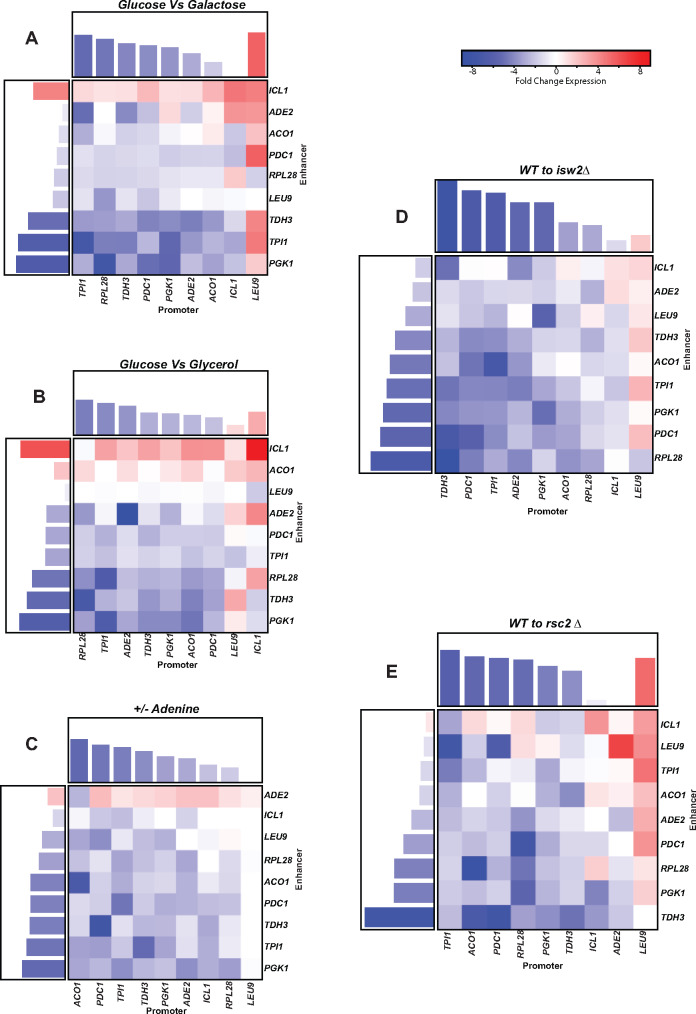
Expression analysis of the 9** × **9 matrix of UAS enhancers and core promoters + 5′ UTR under varying growth conditions. Cells containing the 81 combinations of 9 UAS enhancers with 9 core promoters + 5′ UTR were grown in different growth conditions and expression of the Venus cassette was measured in a fluorometer. Three biological replicates were measured for each construct. (**A**) The difference in expression of cells grown in medium containing glucose to cells grown in medium containing galactose are plotted as heat maps. Bar graphs above and on the left of the heat map are a summation of the nine individual values. (**B**) The difference in expression of cells grown in medium containing glucose to cells grown in medium containing glycerol are plotted as heat maps. Bar graphs above and on the left of the heat map are a summation of the nine individual values. (**C**) The difference in expression of cells grown in medium containing adenine to cells grown in medium lacking adenine are plotted as heat maps. Bar graphs above and on the left of the heat map are a summation of the nine individual values. (**D**) The difference in expression of wild-type cells grown in medium containing glucose to *isw2Δ* cells grown in medium containing glucose are plotted as heat maps. Bar graphs above and on the left of the heat map are a summation of the nine individual values. (**E**) The difference in expression of wild-type cells grown in medium containing glucose to r*sc2Δ* cells grown in medium containing glucose are plotted as heat maps. Bar graphs above and on the left of the heat map are a summation of the nine individual values.

The 81-cassette matrix can be easily adapted to study gene activation in different growth conditions (Figure 9). To demonstrate the versatility of this system, we chose to study expression in galactose- and glycerol-containing media since galactose is a fermentable sugar while glycerol is a non-fermentable carbon source. Comparison of the cells grown in glucose-, galactose- and glycerol-containing media (as well as media lacking adenine) shows the role of UAS enhancers and core promoters in integrating environmental cues such as a change in carbon source as well as nucleotides. For example, when comparing changes in gene expression in glucose compared to galactose-containing media, we find that the *ICL1* enhancer becomes derepressed in galactose. Similarly, in glycerol-containing media both the *ICL1* and *ACO1* UAS enhancers become active while genes involved in fermentation show reduced activity. The same analysis done in media containing or lacking adenine shows a similar effect for the *ADE2* UAS enhancer.

We also performed this comparative analysis in mutants for chromatin remodeling factors Rsc2 and Isw2. Both of these proteins play a role in organizing the NFR at the core promoter as well as the −1 and +1 nucleosomes flanking the NFR. In an Rsc2 mutant the fold expression of most genes is reduced (Figure 9) though the opposite effect is observed at the *LEU9* promoter suggesting that the repressive effects of the *LEU9* promoter may be Rsc2 dependent possibly via an unfavorable placement of nucleosomes over the core promoter. The same change in expression is observed in an Isw2 mutant. These data seem to suggest that at most promoters, chromatin remodelers are required for gene activation while at the *LEU9* promoter the repressed state is maintained by a specific nucleosome configuration that is weakened in the absence of the chromatin remodeling proteins.

## 3. Discussion

Transcription is a result of transcription factor binding at the UAS enhancer and effective communication with proteins bound to the core promoter (28). The permutational analysis of a variety of UAS enhancers and promoters rapidly provides a panoramic view of functional interactions between these elements.

The data reveal specific patterns of expression mediated by different combinations of enhancers and promoters. For example, our expression matrix reveals that the *RPL28* and the *CDC19* UAS enhancers have distinct expression patterns when paired with the different core promoters compared to the patterns observed with other glucose-induced enhancers (such as *TDH3, PGK1, PDC1*). The *RPL28* and *CDC19* genes are regulated by the transcription activators Rap1p and Abf1p. Rap1p binds 300–400 bp upstream from the transcription start site and has the ability to evict nucleosomes ∼400 bp from its binding site (29–32). On the other hand, genes required for growth in glucose-containing media (such as *TDH3*, *PGK1*, *PDC1*) are regulated in part by the transcription activators Reb1p and Gcr1p, which bind near the −1 nucleosome and promote RSC-mediated nucleosome mobility immediately downstream of their binding sites (29, 30, 32, 33). It is possible that the ability of Rap1p to mobilize nucleosomes over a greater distance translates into its ability to activate genes from a more diverse set of core promoters. A fuller understanding of the mechanisms underlying these differences will require further mutagenic and molecular analysis of these synthetic constructs.

Our expression analyses show that the core promoters affect expression levels independent of the transcription factors bound to the UAS enhancers. This suggests that one function of the core promoter is to act as an integrator of signals emanating from the UAS enhancer and a modulator of expression levels. In yeast, there are two core promoter architectures—TATA-containing core promoters and TATA-less core promoters (3, 5, 21, 34–36). Our data suggest that the presence of a TATA box is likely to increase the levels of transcripts produced by an enhancer. The underlying molecular mechanism is most likely modulation of TBP binding. The presence of a TATA box at a core promoter likely increases the probability of the formation of a functional pre-initiation complex at the promoter since TATA boxes are high-affinity binding sites for TBP/TFIID (21). Thus, weak activators stimulate transcription via a molecular mechanism that benefits from the enhanced affinity of Transcription factorIID (TFIID) binding to the core promoter while strong activators can mediate high levels of transcription even in the presence of a sub-optimal core promoter.

Another aim of these experiments was to generate synthetic regulatory elements that exhibited varying activity levels similar to approaches previously used to explore enhancer–promoter combinations (1, 37, 38). Using this approach, we have identified combinations of regulatory elements that generate a larger spectrum of activity than the native element. These synthetic cassettes, where the same enhancer is coupled with different core promoters, allow one to change expression levels without significantly altering the ability of the cassette to respond to external stimuli could be a useful resource.

## 4. Materials and methods

### 4.1 Golden gate cloning

All regulatory fragments were PCR amplified from S288c genomic DNA using specific primer pairs. Each fragment was amplified with primers containing a BsmBI recognition site that upon digestion would create sticky ends with the sequences TCGG and GACC at the 5′ and 3′ ends, respectively. Adjacent to the BsmBI site, each PCR primer also contained a sequence that is recognized by BsaI and that upon digestion would create specific sticky ends for each regulatory element. Thus, the enhancer fragments had BsaI sticky ends with the sequences AACG and TGGC while the core promoter fragments had BsaI sticky ends with the sequence TGGC and TTCT. The 5′ UTR fragments had BsaI sticky ends with the sequence TTCT and TATG, the mRuby2 reporter gene fragment had BsaI sticky ends TATG and ATCC while the 3′ UTR fragments had BsaI sticky ends with the sequences ATCC and GCTG. The TGGC sequence in the promoter fragment was followed by the sequence TATGCC. During assembly, the 6-bp insertion along with the 4-bp TGGC Golden Gate scar in effect inserts a 10-bp fragment between the upstream enhancer and the TATA box. A 10-bp insertion was chosen at this site since 10 bp insertions between the enhancer and the TATA box has previously been shown to be optimal for transcription while 5 bp insertions are deleterious for optimal transcription (5).

The amplified DNA fragments were cleaned using a Bioline PCR purification kit. Purified PCR products were quantified using a Nanodrop spectrophotometer and cloned into pYTK001 (9) with the enzyme BsmBI. Sixty fmoles of insert were combined with 60 fmoles of plasmid DNA along with 1**×** T4 DNA ligase buffer, bovine serum albumin (BSA), 1 μl BsmBI (New England Biolab (NEB)) and 1 μl high concentration T4 DNA ligase (NEB). The reaction was incubated for 50 cycles at 37°C for 3 min, and at 16°C for 4 min. The reaction was terminated by incubating at 50°C 5 min followed by 80°C 5 min and stored at 4°C until ready to use.

Between 1 and 2 μl of each ligation reaction was transformed into 25 μl of DH10B competent cells and plated on 2xTY plates containing chloramphenicol. Between two and five colonies were picked, grown overnight in selective media and plasmid was isolated using a Qiagen mini-plasmid purification kit. Plasmids were checked for inserts using insert specific primers.

The 103 parts plasmids (we were unable to clone the *HIS3* core promoter) along with mRuby2 plasmid were then used to create a combinatorial library such that the different fragments would combine in the correct order but in a random manner. To join the different regulatory fragments, we mixed 30 fmoles of each parts plasmid containing the different regulatory fragments with 30 fmoles of vector [an *ARS/CEN/URA3* derivative of pYTK096 (9)] in the presence of the enzyme BsaI in a Golden Gate reaction as described above. Multiple reactions were performed in parallel and pooled prior to *Escherichia coli* transformation. DH10B competent cells were electroporated with the library and transformed cells were plated on multiple large 2xTY plates containing Kanamycin. Cells were scrapped off the plates, and plasmid DNA was isolated from these cells and purified on a Cesium Chloride gradient.

A 1**×** coverage of all possible permutations (26** × **25** × **26** × **26) would result in 439 400 clones and we obtained over 600 000 *E. coli* colonies. Based on random sampling (39), we therefore expect 74% of the possible clones to be present in this library at least once.

### 4.2 Yeast electroporation of library

Yeast strain (ROY5634: *MATa ADE2 lys2D leu2-3,112 his3-11 ura3-1 trp1-1 can1-100 rpl18b::BFP2-2*) was transformed with the library DNA (40). Cells were grown overnight in 5 ml Yeast extract-Peptone-Dextrose (YPD) medium. Two hundred milliliters fresh YPD was inoculated with the overnight culture at a concentration of 0.25 OD/ml and grown for 5 h. Cells were spun and resuspended in 50 ml YPD with Tris:DTT and incubated at 30°C for 30 min with shaking. Cells were washed with 25 ml Buffer-E (10 mM Tris–HCl pH 7.5, 270 mM Sucrose, 1 mM MgCl_2_) and resuspended in 2 ml Buffer-E DNA was added to the cells and 100 μl of cells were placed in a 0.2-mm cuvette and electroporated at 540 V 25 μF, infinite resistance with an exponential pulse. Cells were resuspended with YPD, incubated at 30°C for 1 h without shaking and then plated on YMD plates lacking uracil. After 3 days, ∼10 000 colonies were present on each plate. Greater than 200 000 yeast transformants were scraped off the plate into YMD lacking uracil media, grown for 5 h at 30°C and then frozen at −70°C in the presence of 20% glycerol.

### 4.3 Cell sorting

Five milliliters of yeast cells containing the library were used to inoculate 50 ml YMD medium containing adenine, leucine, lysine, histidine and tryptophan (lacking uracil) and cells were grown at room temperature overnight (∼3 doublings). This culture was then used to inoculate 250 ml YMD-uracil and grown at 30°C for 3 h. Cells were pelleted and resuspended into 250 μl 1**×** phosphate buffered saline and 1% BSA at a concentration of 1 OD/ml, filtered through a Nitex mesh and sorted.

The gates for fluorescent cell sorting were based on various control strains. Prior to sorting the library, we analyzed four different transformants, a strain that did not express mRuby2 or mTagEBFP2-2, a strain that only expressed mTagEBFP2-2, a strain that only expressed mRuby2 and a strain that highly expressed mRuby2 along with mTagEBFP2-2 (Supplementary Figure S1). Based on the mRuby2 and mTagEBFP2-2 fluorescence distributions of these four strains, we designed gates that would sort the cells into four expression fractions—no mRuby2 expression, low, medium and high mRuby2 expression relative to constitutively expressed mTagEBFP2-2 and cells not meeting these criteria were not collected. We then sorted the yeast cells containing the library and collected a total of 24 540 211 cells (Figure 1).

### 4.4 Insert library preps from sorted cells

Total DNA was prepared from the unsorted library, no expression sorted fraction (2.36** × **10^7^ cells), low expressing sorted fraction (19.5** × **10^6^ cells), medium expressing sorted fraction (17.1** × **10^6^ cells) and high expressing sorted fraction (6** × **10^6^ cells) using the YeaStar Genomic DNA kit from ZymoResearch. Two hundred nanograms of Nanodrop quantitated DNA from each fraction were treated with ExoVII and Exo VIII (Truncated) (NEB) to reduce the amount of linear genomic DNA. Following denaturation of the enzymes at 80°C for 10′, inserts in the plasmid library fractions were amplified with primers using the KAPA HiFi PCR kit in multiple 25 μl reactions (10×–50×). Reactions were pooled and precipitated with glycogen and ethanol and the precipitated DNA was resuspended in nuclease-free water. AmPure XP beads (0.8 vol) were used to purify DNA >700 bp from the sorted insert libraries. This DNA was quantitated using the Qubit Broad Range DNA kit and subsequently analyzed by conventional agarose and BioAnalyzer gel electrophoresis and eventually used for sequencing.

### 4.5 Oxford Nanopore sequencing

Seven hundred nanograms of DNA from each of the no, low, medium and high expressing sorted insert libraries were used to prepare samples for sequencing on the Oxford Nanopore MinION. Nanopore barcodes (NB01 > NB04) were individually ligated to each end-repaired and dA tailed fraction, which was quantitated and pooled in specific ratios prior to adapter ligation. Adapters were ligated to the pooled barcoded libraries according to the Oxford Nanopore protocol. The DNA was then quantitated and loaded on the nanopore flow cell for sequencing. We obtained a total of 1 662 773 reads from the four sorted fractions that mapped to the 26 gene fragments. Seventy-three percent of the mapped reads had all four regulatory fragments in the correct order while the remainder lacked a fragment either due to inappropriate joining during ligation or due to our inability to unambiguously assign an identity to the fragment after sequencing. Some of the fragments were absent in the sequences obtained. The *HIS3* core promoter was absent since we were unable to clone this part. Also, the *PHO5* and *ADH1* enhancer fragments were missing because these fragments had a BsaI site within the fragment. This likely precluded these two fragments from being ligated together during the library preparation. Finally, for reasons not quite clear, we observed a higher than expected presence of the *ADH1* 3′ UTR in the library.

### 4.6 Microtiter plate transformations of yeast cells

Yeast strains were grown for 6 h at 30°C in 5 ml YPD with shaking. Four hundred milliliters YPD was inoculated with these cells so that the final concentration of cells after 14 h of growth is 2 OD/ml. Three hundred milliliters of cells were pelleted, washed in 150 ml 0.1 M lithium acetate and resuspended in 3 ml 0.1 M lithium acetate. Three hundred and thirty-three microliters sonicated salmon sperm DNA (10 mg/ml) was added to the cells. In each well of a microtiter plate 3–5 μl plasmid DNA was added. Twenty-five microliters of yeast cells were mixed with the plasmid DNAs in the microtiter plates and the cells were incubated at 30°C for 15 min. One hundred and fifty microliters Polyethylene glycol/Lithium acetate was added to each well and mixed by pipetting. The cells were further incubated for 30 min at 30°C. Seventeen microliters Dimethylsuffoxide (DMSO) was added to the cells and the plates were heat shocked at 42°C for 15 min in a heat block. Cells were spun and supernatant was aspirated off. Cells were resuspended in 10 μl water and plated onto plates (Yeast minimal media dextrose (YMD) lacking uracil). Colonies were allowed to grow for 3 days at 30°C (41).

### 4.7 Fluorescence measurements using a plate reader

Transformed yeast cells were transferred from a plate using a frogging tool (Sigma) into three different microtiter plates each containing 100 μl YMD-uracil media. Cells were grown overnight at 30°C without shaking. Thirty microliters of each culture was used to inoculate deep well (2 ml) microtiter plates containing 570 μl YMD-uracil. Cultures were grown overnight at 30°C with shaking at 600 rpm. Thirty microliters of these overnight cultures were used to inoculate fresh 2 ml microtiter plates containing 570 μl YMD-uracil and grown overnight at 30°C with shaking. One hundred microliters of fresh YMD-uracil media in 2 ml microtiter plates were inoculated with 50 μl overnight cultures and grown at 30°C for 3 h with shaking. One hundred microliters of each culture was removed and fluorescence was measured using a microtiter fluorescent plate reader.

### 4.8 Determination of expression in sorted yeast cells

To determine the mean and variance of expression for a regulatory fragment we fit an estimate of that fragment’s prevalence in each fraction to a log-normal model of protein expression, as described (42). The estimate, $x_{i,b}$, of the number of cells containing fragment, i, sorted into each fraction, b, was determined by normalizing the number of reads,$\;r_{i,b}$ by multiplication with $\frac{C_{b}}{R_{b}\sum C_{b}}$, where $C_{b}$ and $R_{b}$ are the total number of cells sorted and reads mapped from bin b, respectively (effectively calculating the fractional representation of fragment, i, in bin b and subsequently scaling that fraction by the fraction of cells observed in bin b by FACS). We then assume that $x_{i,b}\;$are random variables sampled from binned log-normal distributions where the bins are determined by the FACS fraction boundaries

$$x_{i}=\left[\begin{array}{c} \int_{\log(A_{3})}^{+\infty }N(x,\;\log{(\mu }_{i}),\sigma_{i})\\ \int_{\log(A_{2})}^{\log(A_{3})}N(x,\;\log{(\mu }_{i}),\sigma_{i})\\ \int_{\log(A_{1})}^{\log(A_{2})}N(x,\;\log{(\mu }_{i}),\sigma_{i})\\ \int_{-\infty }^{\log(A_{1})}N(x,\;\log{(\mu }_{i}),\sigma_{i}) \end{array}\right],$$

where$\;x_{i}$ is the vector of ratios for all bins described above, $\mu_{i}$ is the mean expression, $\sigma_{i}$ is the standard deviation of expression and $A_{b}$ is the expression value for the upper boundary of bin *b* by FACS.

## Reagents and data sharing 

All plasmids and strains developed for this study will be available upon request. All sequencing data have been deposited and will be available at https://www.ncbi.nlm.nih.gov/sra/PRJNA608939. All scripts used to analyze the data are available at https://github.com/rshelans/CombiAnalysis.

## Funding

This work was supported in part by a grant from the National Institutes of Health (NIH) [GM078068 to R.K.]. M.J. was supported on an National Institutes of Health (NIH) grant to M. Akeson [HG007827].

## Supplementary Material

ysaa007_Supplementary_Data
